# A single-center retrospective comparison of pT1 substaging methods in bladder cancer

**DOI:** 10.1007/s00428-024-03907-4

**Published:** 2024-09-02

**Authors:** Johannes Kläger, Maximilian C. Koeller, André Oszwald, Gabriel Wasinger, David D’Andrea, Eva Compérat

**Affiliations:** 1 Department of Pathology, Medical University of Vienna, Vienna, Austria; 2 Department of Urology, Medical University of Vienna, Vienna, Austria

**Keywords:** Anatomical substaging, Interobserver variability, Metrical substaging, T1 urothelial cancer, T1 urothelial cancer substaging, Urothelial carcinoma, Staging urothelial carcinoma

## Abstract

Substaging of T1 urothelial cancer is associated with tumor progression and its reporting is recommended by international guidelines. However, it has not been integrated in risk stratification tools and there is no agreement on the best method to use for its reporting. We aimed to investigate the applicability, interobserver variability, and prognostic value of histological landmark based and micrometric (aggregate linear length of invasive carcinoma (ALLICA), microscopic vs. extensive system, Rete Oncologica Lombarda (ROL) system) substaging methods. A total of 79 patients with the primary diagnosis of T1 urothelial cancer treated with conventional transurethral resection and adjuvant BCG therapy between 2000 and 2020 at the Medical University of Vienna were included. The anatomical and metrical substaging systems were evaluated using agreement rate, Cohen’s kappa, Kendall’s tau, and Spearman rank correlation. Prognostic value for high-grade recurrence or T2 progression was evaluated in uni- and multivariable analysis. Applicability and reproducibility were good to moderate and varied between substaging methods. Obstacles are mainly due to fragmentation of samples. Anatomical substaging was associated with progression in univariable and multivariable analysis. In our cohort, we could only identify anatomical landmark–based substaging to be prognostic for T2 progression. A major obstacle for proper pathological assessment is fragmentation of samples due to operational procedure. Avoiding such fragmentation might improve reproducibility and significance of pathological T1 substaging of urothelial cancer.

**Supplementary Information:**

The online version contains supplementary material available at 10.1007/s00428-024-03907-4.

## Introduction

The standard treatment of T1 urothelial carcinoma (UC) of the bladder is complete transurethral resection (TURB) followed by adjuvant intravesical instillation with BCG. However, despite adequate treatment, roughly half of patients will experience disease recurrence and 20% progression to invasive disease [1, 2]. Given the potential high aggressiveness of this disease in some cases, the risk of both under- or overtreatment is high. Significant efforts have been made to develop risk stratification tools and identify biomarkers that can accurately predict which patients are likely to fail BCG therapy and would benefit from an upfront radical cystectomy [3]. However, none of these biomarkers could achieve a sufficiently high discrimination to be implemented in clinical practice and guide decision-making, leaving the pathological stage and grade as the main driver.

In this context, pathologic substaging of T1 disease has been investigated as an option to better stratify patients. This involved the evaluation of the invasion extent either according to anatomical landmarks using a two- or three-tiered system, or based on micrometric evaluation of the invasion often using a defined cut-off in a two-tiered system [4, 5]. Despite the growing bulk of evidence, T1 substaging is still not part of current risk stratification tools. This is partially attributable to the lack of comparative studies and validation in external cohorts [1, 4–7].

To fill this gap in knowledge, we investigated the differential applicability, interobserver variability, and prognostic value of histological and micrometric T1 substaging methods.

## Materials and methods

### Patient population

Patients with a primary diagnosis of T1 UC of the bladder were eligible for inclusion. Patients were treated with transurethral resection (TURB) and subsequent BCG therapy between 2000 und 2020 at the Medical University of Vienna. A second look TURB was performed in 80% of patients within 6 weeks after the diagnosis of T1 UC. Patients with previous diagnosis of UC and patients with upper tract UC were excluded.

### Tissue-based analyses

Archived H&E slides from formalin-fixed and paraffin-embedded tissue samples of each case were retrieved. Cases for which no histological slides were available and cases that did not show invasion during re-evaluation were excluded. Other systematically evaluated pathological features, such as lymphovascular invasion or histological subtype, were retrieved from initial pathology reports. All slides were scanned as whole slide images with a Pannoramic 250 III Flash scanner (3DHistech, Budapest, Hungary) at a resolution of 0.243 µm/px, and subsequent histological examination and substaging were performed using the Cytomine platform (Cytomine Corporation SA, Liège, Belgium, v4.3.5-beta) [8].

Anatomical landmark-based substaging was defined as T1a (invasion not involving the muscularis mucosa (MM)) and T1b (invasion involving or beyond MM). In the case of absence of MM, the vesicular vascular plexus (VVP) was used as surrogate. Cases that could not be classified as T1a or T1b by anatomical substaging were designated as T1 and excluded from further analyses. For micrometric-based substaging, the number of invasive foci was recorded and the maximum diameter of every invasive focus was measured irrespective of the orientation of this diameter in regard to the urothelial surface. The measurements on the digitalized slides were done using Cytomine [8] and QuPath [9] version 0.4.3. The aggregate linear length of invasive carcinoma (ALLICA) was then calculated as the sum of the diameter of invasive foci in millimeters. Based on focality and ALLICA, samples were categorized as using both, the “microscopic vs. extensive” system and the Rete Oncologica Lombarda (ROL) system [10, 11]. In short, the “microscopic vs. extensive” system classifies a case as microscopic (“m”) in the case of a single focal invasion of ≤ 0.5 mm, and otherwise as extensive (“e”). The ROL system classifies a case as ROL1 if the sum of all invasive foci is ≤ 1 mm, irrespective of the number of invasive foci, and otherwise as ROL2. For systematic display of applied methods, see Table 1.

**Table 1:**
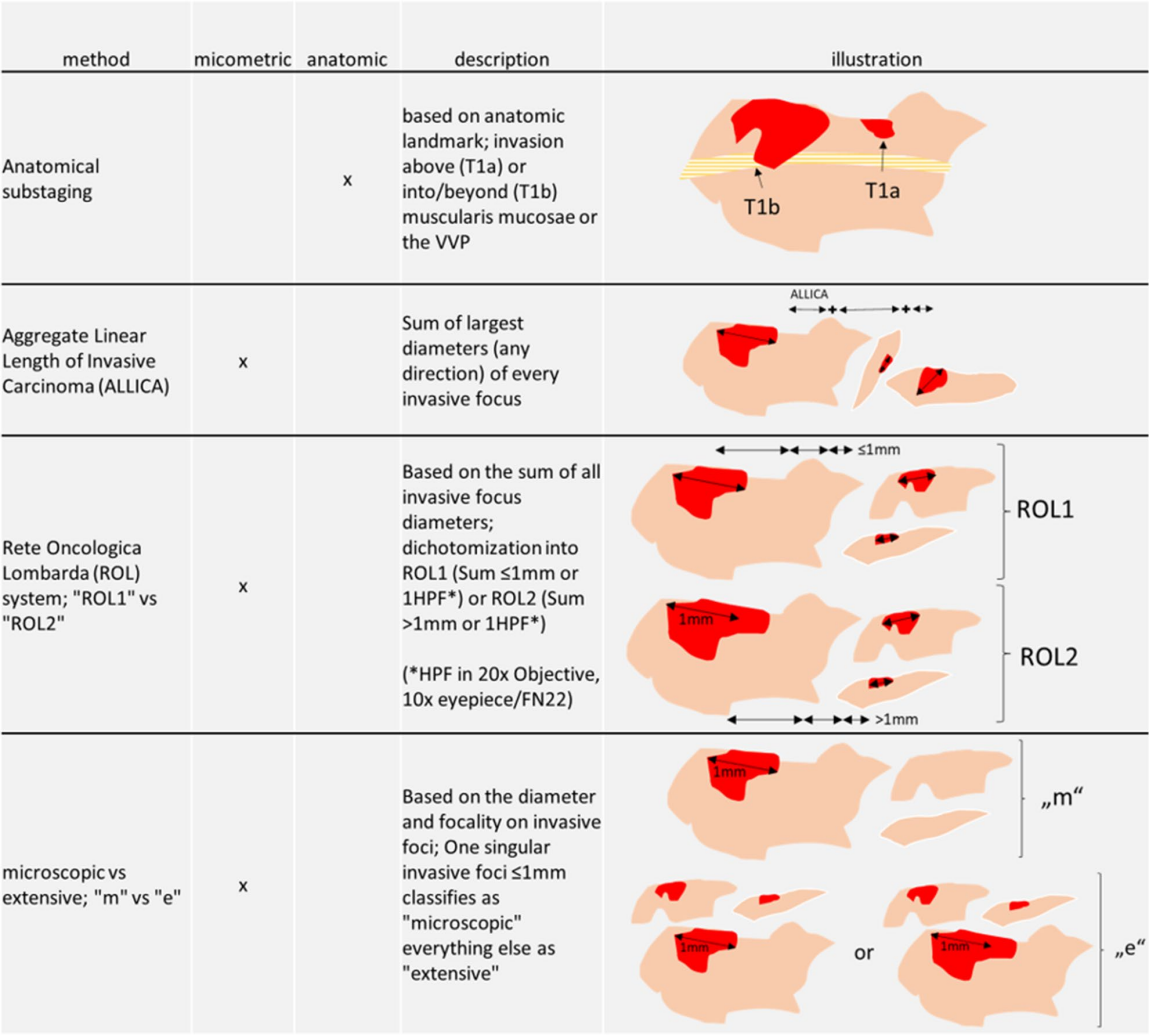
Descriptive and schematic display of substaging methods used in this analysis; *VVP* vesicular vascular plexus

All slides were re-reviewed for extent of lamina propria invasion separately by two dedicated uropathologists, blinded to each other and to the oncologic outcomes of the patient. Discrepancies in anatomical landmark–based substaging were subsequently discussed to reach a consensus result. This consensus result was used for subsequent analyses. For measurement-based substaging, no consensus measurements were made but results of both observers were used for subsequent analysis.

Our primary endpoint was reproducibility and applicability of different histology-based substaging methods. Secondary endpoints were prognostic value of substaging methods for the progression rate to T2 urothelial cancer and high-grade (HG) recurrence within 3 years after initial diagnosis. Patients who died without progression or were lost to follow-up were censored at the date of death or last follow-up visit.

Statistical analysis was done using SPSS version 25 (IBM). Group comparison was done using fisher exact test for categorical variables and Mann–Whitney *U* test for metrical variables. Interobserver agreement was assessed using agreement rate and Cohen’s kappa for categorical classification systems and Spearman’s correlation coefficient and Kendall’s tau for ALLICA. Progression and recurrence rate was assessed using uni- and multivariable Cox regression analysis. Receiver operating characteristic (ROC) curve was used for visualization and evaluation of a potential binary classifier based on ALLICA measurements.

The study was approved by local ethics committees and is in line with the declaration of Helsinki and its revisions.

## Results

Our cohort consisted of 79 patients with de novo diagnosis of T1 urothelial cancer, who were all treated with TURB and consecutive BCG instillation. Clinical and pathological baseline data at the time of TURB is summarized in Table 2. The gender ratio (male to female) was 62:17 and the median age was 69 years (IQR 63–78) with no significant age difference between gender (*p* = 0.56). Histologically, all lesions were high-grade according to WHO grading and mostly conventional UC (93.7%). Lymphovascular invasion was present in 13 cases and carcinoma in situ (Cis) in 37. Detrusor muscle (muscularis propria) was present in 69 cases.

**Table 2:** Baseline demographic and histological data at time point of initial diagnosis

Demographic data		Total		
Gender		79	17 (female)	62 (male)
Age [a] (median, IQR)		69 (63–78)	76 (60–81)	69 (63–76)
Smoker	Ever	34	4	30
	Never	11	6	5
	Missing	34	7	27
Clinical tumor size	Length (cm) (median, IQR)	3 (2–4)	3 (2–4)	3 (2–5)
	< 3 cm	30	7	23
	≥ 3 cm	49	10	39
BCG treatment	No	0	0	0
	Yes	79	17	62
Irrigation NaCL	No	5	0	5
	Yes	74	17	57
MMC single-shot	No	67	17	50
	Yes	12	0	12
Histological data		Total	Female	Male
Grade (WHO)	Low grade	0	0	0
	High grade	79	17	62
Carcinoma in situ	No	42	7	35
	Yes	37	10	27
Histology subtype	Conventional UC^a^	74	16	58
	Micropapillary UC^a^	2	1	1
	Squamous UC^a^	1	0	1
	Poorly differentiated UC^a^	^a^2	0	2
Lymphovascular invasion	No	66	11	55
	Yes	13	6	7
Muscularis propria present	No	10	2	8
	Yes	69	15	54

^a^Urothelial cancer

The median observational time was 34 months (IQR, 14–36), in which cancer-related death occurred only in one patient (six patients died of cancer-unrelated cause). Eleven patients (13.9%) showed progression to muscle invasive carcinoma; 25 (31.6%) patients had HG recurrence. In the group of patients who progressed to T2 cancer, the median time until progression was 8 months (IQR 5–24). In univariable analysis, none of our baseline characteristics were associated with progression rate and only presence of Cis was associated with HG recurrence (Supplementary Table 1).

Interobserver agreement of anatomical landmark–based substaging was moderate with Cohen’s kappa of 0.63 with 16 discrepancies corresponding to an agreement rate of 79.7% (Table 3). Consecutive consensus scoring was done for discrepant cases. Overall, six cases could not be categorized as either T1a or T1b (7.6%) due to insufficient specimen orientation leading to inability to access infiltration depth properly. These cases were categorized as T1 and excluded from further analyses. Anatomical substaging was not associated with HG recurrence (*p* = 0.78) but with T2 progression (*p* = 0.03) and was prognostic for progression-free survival in univariable analysis (HR 4.26; CI 95% 1.10–16.48; *p* = 0.083) and also in multivariable analysis including age, gender, LVI, CIS, histologic subtype, tumor size > 3 cm, and presence of muscularis propria (HR 6.89; CI 95% 1.20–39.50; *p* = 0.030) (Table 4 and suppl.Table 2).

**Table 3:** Interobserver variabilty of T1 substaging methods

			Observer2				Agrrement rate	Cohens kappa	Pearson's coefficient	Kendall's tau
AS		Observer1		T1a	T1b	T1	79.75%	0.63		
			T1a	40	6	1				
			T1b	2	20	4				
			T1	3	0	3				
ALLICA	"m" vs. "e"			m	e			0.37		
			m	3	3		89.90%			
			e	5	68					
	"ROL1" vs. "ROL2"			ROL1	ROL2			0.65		
			ROL1	10	4		89.90%			
			ROL2	4	61					
	mean lenght [mm]	Observer 1	14.2 (SD=25.0)						0.81 (*p* < 0.01)	0.69 (*p* < 0.01)
		Observer 2	11.0 (SD=14.0)

**Table 4:** Prognostic value of T1 substaging methods for high grade recurrence and T2 progression

			HG rec					T2 progression				
			Yes	No	Total	*p*	Cox regression univariable	Yes	No	Total	*p*	Cox regression univariable
	AS consensus score	T1a	34	16	50	0.60	HR 1.13 (CI95% 0.49–2.60 *p*=0.78)	45	18	63	0.03	HR 4.55 (CI95% 1.18–17.59 *p*=0.03)
		T1b	14	9	23			3	7	10		
		T1			6					6		
Observer 1	ALLICA (median, IQR) [mm]		6.71 (2.51–16.03)	7.30 (1.96–17.88)	7.30 (2.39–17.44)	0.85	HR 0.99 (CI95% 0.97–1.01 *p*=0.49)	7.45 (2.40–16.03)	7.18 (2.35–17.84)	7.30 (2.39–17.44)	0.87	HR 0.99 (CI95% 0.95–1.03 *p*=0.53)
	"m" vs. "e"	m	2	6	8	> 0.9	HR 0.78 (CI95% 0.18–3.32 *p*=0.74)	1	7	8	> 0.9	HR 1.0 (CI95% 0.13–7.70 *p*=0.99)
		e	23	48	71			10	61	71		
	"ROL1" vs. "ROL2"	ROL1	4	10	14	> 0.9	HR 0.92 (CI95% 0.31–2.67 *p*= 0.87)	1	13	11	0.68	HR 2.23 (CI95% 0.29–17.42 *p*= 0.45)
		ROL2	21	44	65			10	55	63		
Observer 2	ALLICA (median, IQR) [mm]		8.68 (2.61–19.92)	4.43 (1.65–18.13)	5.47 (1.90–18.69)	0.30	HR 1.01 (CI95% 0.99–1.03 *p*=0.45)	6.65 (2.37–19.56)	5.07 (1.84–18.60)	5.47 (1.90–18.69)	0.81	HR 0.99 (CI95% 0.94–1.04 *p*=0.57)
	"m" vs. "e"	m	2	4	6	> 0.9	HR 1.11 (CI95% 0.38–3.22 *p*=0.86)	1	5	6	> 0.9	HR 1.02 (CI95% 0.22–4.74 *p*=0.98)
		e	23	50	73			10	63	73		
	"ROL1" vs. "ROL2"	ROL1	4	10	14	> 0.9	HR 1.11 (CI95% 0.38–3.22 *p*= 0.88)	2	12	14	> 0.9	HR 1.02 (CI95% 0.22–4.74 *p*= 0.97)
		ROL2	21	44	65			9	56	65		

ALLICA measurements of invasive foci showed good correlation between observers (Pearson’s coefficient 0.81; *p* < 0.01, Kendall’s tau 0.69; *p* < 0.01) and could be applied in all cases (Table 2). Stratification based on ALLICA measurements into “m” vs. “e” and “ROL1” vs. “ROL2” as described in the literature revealed moderate interobserver agreement for the m/e system (Cohen’s kappa 0.37; agreement rate 89.9%) and better agreement for the ROL system (Cohen’s kappa 0.65; agreement rate 89.9%) (Table 3). For micrometric-based methods, no consensus score or consensus stratification was obtained. Consequently, the following analyses were performed twice and *p*-values are displayed for both observers. Overall, micrometric stratification did not demonstrate significant correlation with progression or recurrence, neither using the “m vs e” system (*p* > 0.99 both observers) nor using the ROL system (*p* = 0.68 and *p* > 0.99) (Table 4). There was no significant difference between lengths of ALLICA in relation to disease progression (*p* = 0.87 and *p* = 0.81) (Table 4), and ROC analysis revealed AUC values close to 0.5 (0.53 respectively 0.51, Supplementary Fig. 1). It was concluded that—in this dataset—useful cut-off value for micrometric substaging could be determined.

## Discussion

### Applicability and reliability

**Fig. 1:**
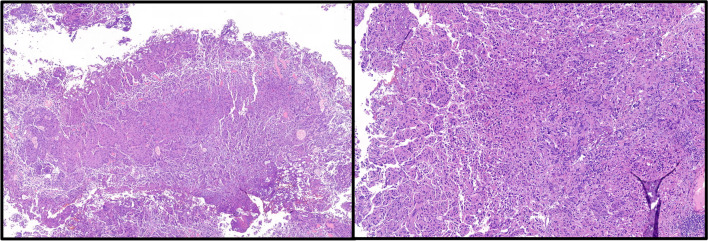
Tangentially cut cTURB fragments with invasive urothelial cancer, rendering it impossible to discern depth of invasion in relation to potential muscularis mucosa or vesicular vascular plexus

**Fig. 2:**
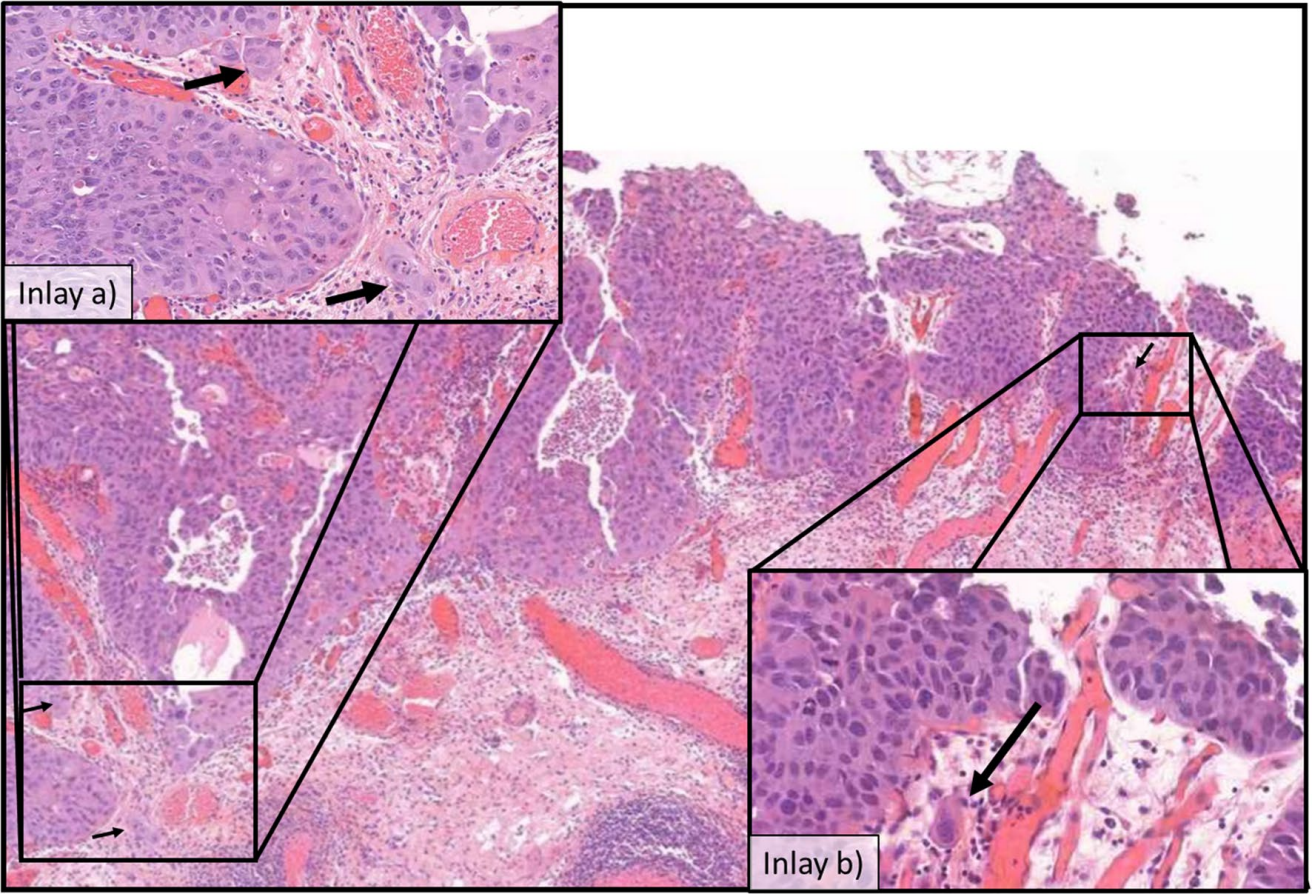
Illustrating problems arising in micrometric and focality-based substaging methods. **a** Two invasive foci in proximity—are they to be counted as separate or singular? **b** A singular invasive cell, but probably critical in evaluation of single vs. multifocality

Anatomical substaging can be applied in most cases; however, six (8%) cases remained as unclassifiable due to insufficient specimen orientation resulting in tangentially cut invasive foci in which no MM or VVP can be identified (Fig. 1). Other studies assessing AS reported applicability for 40.8–100% of cases with reasons for inapplicability, if reported, like cutting artifact, impaired orientation, or absence of defined anatomical landmark [11–16]. Additionally, the VVP as a widely used surrogate for the MM is inaccurately defined and may extend throughout the thickness of the lamina propria [17]. Substaging based on a micrometric approach is reported to be more feasible, resulting in applicability rates of 96.0–100% [11, 12, 16, 18, 19]. Most of these obstacles are conditional by the procedure of TURB [20, 21]. En bloc resection might be an alternative operational procedure to enhance specimen orientation and thereby applicability, accuracy, and reliability of T1 substaging, especially, but not exclusively, for anatomical landmark-based methods [21–23]. Only limited data is available concerning reliability, e.g., interobserver variability. Most of the studies investigating T1 substaging do not explicitly report on this issue, even though in most cases more than one pathologist contributed to histology reading [19, 24–26]. Some data is available for assessment of Ta vs. T1, reporting concordance rates of 72–80%, suggesting impaired interobserver agreement between pathologists also in regard to extent of invasion and interconnected substaging [27, 28]. Other studies also reported substantial up- or down-staging of T1 cases that were evaluated for further substaging [11, 26, 29]. Data specifically for T1 substaging is only reported by Grobet-Jeandin et al. and Colombo et al. who reported an agreement rate of 89% for anatomical substaging and an agreement rate of 96% for ROL1/ROL2 substaging, but did not report a kappa value [11, 14]. These results are basically in line with our results; however, due to missing kappa statistics, a comparison is limited. Surprisingly low interobserver agreement was seen for “m” vs. “e” in our evaluation. This is probably caused by the lack of consensus on how far two foci must be apart to be separate (Fig. 2, inlay a) and the problem that also a single invasive cell, representing an additional invasive focus, might cause upstaging to “e” (Fig. 2, inlay b). The issue of perceiving invasive tumor areas proximal to each other as one or more separate foci is also a problem for systems assessing the largest as well as the sum of invasive foci, as one examiner might include “tumor-free” areas between the foci in the measurement, while another might exclude them. Moreover, the necessity of assessing multiple foci in a highly fragmented tissue further increases interobserver variability. Small invasive foci, potentially being critical to cause upstaging, might be overlooked by one, but perceived by another examiner, especially in systems like “m vs. e.” Additionally, the axis of the diameter that is measured may vary between observers and cause additional variability in the results.

### Prognostic value

Two large meta-analyses of anatomical substaging reported a significant prognostic value for disease recurrence and progression rate; however, some studies included reported negative results [4, 30]. Notably, also Cis, LVI, tumor size > 3 cm, and multiple tumors were prognostic for recurrence or progression [30]. In a study of 239 patients with T1 tumors, Grobet-Jeandin et al. showed that anatomical substaging remained the only prognostic factor for progression in a multivariable model including Cis and size > 3 cm [14]. Asimakopoulos et al. who used a three-tier-based anatomical substaging, showed only prognostic value in univariable analysis [13]. In contrast, other studies looking at anatomical substaging based on MM invasion could not show prognostic value for tumor progression [15, 29, 31]. Those results are partially in line with our findings, as we also show prognostic value of anatomical substaging for tumor progression in univariable and multivariable analysis including age, gender, LVI, CIS, histologic subtype, tumor size > 3 cm, and presence of muscularis propria, but not for recurrence. A general obstacle that we encountered in anatomical landmark—based substaging is tissue orientation, particularly in tangentially cut invasive foci, and identification of MM or VVP, an issue already reported by others and possibly a major factor for inconsistent results [32].

In regard to micrometric-based substaging methods, there is quite a variability in proposed systems or rather the applied cut off values for dichotomization. One commonly used system is the two tiered “m vs. e” system in which cases with a single invasive focus smaller than 0.5 mm are categorized as “m” and everything else as “e” [10]. Several studies showed prognostic value for this categorization, while De Marco et al., Budina et al., or Colombo et al., in line with our results, did not [10, 11, 13, 15, 16, 25, 29, 31]. However, 0.5 mm is by far not the only cut-off proposed. Leivo et al. suggested a 2.3-mm cut-off for which they could show a 30% false positivity rate in ROC analysis of tumor progression, while Budina et al. suggested 8.915 mm with a 13.3% false positivity rate [16, 31]. Of note, both studies did not report an AUC value. Applying these proposed cut-offs to our data reveals higher false positivity rates of 69–67% and 38–37% for 2.3 mm and 9.3 mm, respectively (suppl. Table 4). In regard to the consequence of radical cystectomy, we would strive for a rather high specificity, respectively, low false positivity rate, probably less than 10%. This would be a cut-off of 35.0 mm (OS1), respectively, 36.5 mm (OS2) in our data, a value only seven, respectively, six patients surpassed. Furthermore, our data (Supplementary Fig. 2) showed that especially in cases with large invasive tumor, the ALLICA measurements deviate more between observers, indicating lower reliability of higher cut-off values, but which in turn are the ones that correspond to lower false positivity rates. The ROL system, which is based on a cut-off value of 1 mm for dichotomization into ROL1 and ROL2 and in contrast to the “m vs. e” system, does not take the number of invasive foci into account, is probably slightly more applicable in daily routine, as 1 mm can be evaluated during microscopic examination without requiring digital microscopy [11]. Notably, this approach was already prospectively evaluated and was found prognostic for progression in a multivariable analysis [19]. However, in our cohort, also, the ROL system was not prognostic for progression or recurrence.

The WHO and the AJCC both recommend substaging of T1 UC, however without a specific method to be used. Similarly, the recent ISUP consensus report acknowledges the value of T1 substaging and advocates its reporting in routine pathology but also does not make a recommendation concerning the method to be used due to lack of consensus [33]. In our study, we could find anatomical landmark–based substaging to be superior and failed to show association with recurrence or progression for micrometric-based substaging methods. Nonetheless, we think that reporting extensiveness additionally to depth of invasion can help in clinical decision-making but potential limitations and shortcomings should be communicated.

Noteworthy limitations of this study are a comparably low case number of 79 patients spanning a time period from 2000 to 2020 and the retrospective design. During these 20 years, management of patients with urothelial cancer changed of course, potentially influencing outcome. However, this cohort consists only of patients who had TURB and consecutive BCG treatment. We also do not have data on distant metastasis, and cancer-specific death occurred only once during our follow-up period of 3 years, making it impossible to draw conclusion concerning risk for metastasis or cancer-specific survival.

Lastly, it must be noted that all our cases were high-grade according to WHO grading. This is in line with the expected low incidence [34]. Together with the reported non-association with outcome, this might be a hint that this grading is probably not useful in the setting of invasive UC [34].

## Conclusion

In this investigation, no classification system could consistently reproduce its significance for recurrence, and only anatomical landmark–based substaging showed prognostic value for T2 progression. Additionally, all systems suffer shortcomings in reproducibility and, to varying degrees, applicability in everyday practice. This is mostly due to the inherent problem of tissue fragmentation and concomitant lack of orientation associated with conventional TURB resection. En bloc resection might be an alternative approach, retaining tumor integrity, thereby improving pathology work-up and concomitantly the assessment of invasion extent–based T1 substaging systems. Future studies investigating invasion extent–based substaging and its prognostic value in en bloc resection specimen are needed to elucidate this hypothesis, which might lead to a more robust histopathology-based substaging system improving patient management and outcome. Nonetheless, until that day, despite all potential shortcomings, we think that reporting of extensiveness and depth of invasion can help in clinical decision-making and should not be omitted but caveats must be communicated.

## Supplementary Information

Below is the link to the electronic supplementary material.

Supplementary file1 (DOCX 83 KB)

## Data Availability

The datasets generated during and/or analyzed during the current study are available from the corresponding author on reasonable request.

## Abbreviations

ALLICA: Aggregate linear length of invasion
BCG: Bacillus Calmette-Guerin
Cis: Carcinoma in situ
TURB: Transurethral resection of bladder cancer
e: Extensive
HG: High grade
m: Microscopic
MM: Muscularis mucosa
OS1/2: Observer 1/2
ROL: Rete Oncologica Lombarda
UC: Urothelial cancer
VVP: Vesicular vascular plexus
