# Supplementary material for: A single-center retrospective comparison of pT1 substaging methods in bladder cancer
Source: Virchows Arch. 2024 Sep 2;486(4):817–26. doi: 10.1007/s00428-024-03907-4 (PMC12018629; doi:10.1007/s00428-024-03907-4)
Supplement: Supplementary file 1 — Supplementary file1 (DOCX 83 KB) [file 428_2024_3907_MOESM1_ESM.docx]

Supplementary table 1: baseline characteristics and their association with high grade recurrence or T2 tumor progresison

| variable |  | total  cohort | high grade  recurrence | | p value | T2 progression | | p value |
| --- | --- | --- | --- | --- | --- | --- | --- | --- |
|  |  |  | no | yes |  | no | yes |  |
| time to event [m] (median IQR) | |  | 7 (5-16) | | | 8 (5-24) | | |
|  |  |  |  |  |  |  |  |  |
| gender | female | 17 | 11 | 6 | 0.77 | 15 | 2 | > 0.9 |
|  | male | 62 | 43 | 19 |  | 53 | 9 |  |
| smoking | never | 11 | 8 | 3 | > 0.9 | 9 | 2 | 0.56 |
|  | ever | 27 | 20 | 7 |  | 25 | 2 |  |
|  | missing | 41 | 26 | 15 |  | 34 | 7 |  |
| age [a] (mean) | | 69.6 | 66.8 | 70.9 | 0.19 | 74.09 | 68.8 | 0.20 |
| clinical tumor size ≥ 3 cm | no | 30 | 22 | 8 | 0.62 | 27 | 3 | 0.52 |
|  | yes | 49 | 32 | 17 |  | 41 | 8 |  |
| Cis | no | 42 | 35 | 7 | <0.01 | 38 | 4 | 0.33 |
|  | yes | 37 | 19 | 18 |  | 30 | 7 |  |
| LVI | no | 66 | 45 | 21 | > 0.9 | 57 | 9 | > 0.9 |
|  | yes | 13 | 9 | 4 |  | 11 | 2 |  |
| irrigation NaCl | no | 5 | 2 | 3 | 0.32 | 5 | 0 | > 0.9 |
|  | yes | 74 | 52 | 22 |  | 63 | 11 |  |
| MMC singleshot | no | 67 | 47 | 20 | 0.50 | 58 | 9 | 0.67 |
|  | yes | 12 | 7 | 5 |  | 10 | 2 |  |
| histological subtype | no | 74 | 51 | 23 | 0.65 | 64 | 10 | 0.53 |
|  | yes | 5 | 3 | 2 |  | 4 | 1 |  |

Supplementary table 2 Multivariable Cox regression analysis of anatomical substaging and selected baseline characteristics

|  | multivariable analysis | | | | | | | |  |  |
| --- | --- | --- | --- | --- | --- | --- | --- | --- | --- | --- |
|  |  |  |  |  |  |  |  |  |  |  |
|  | p value | | HR | | CI 95.0% | | | |  |  |
|  |  |  |  |  |  |  |  |  |  |  |
| anatomical substaging | 0.030 | | 6.89 | | 1.20 | | 39.50 | |  |  |
|  |  |  |  |  |  |  |  |  |  |  |
| age | 0.048 | | 1.08 | | 1.00 | | 1.16 | |  |  |
| gender | 0.464 | | 2.12 | | 0.29 | | 15.71 | |  |  |
| Cis | 0.317 | | 2.08 | | 0.50 | | 8.75 | |  |  |
| LVI | 0.425 | | 0.48 | | 0.08 | | 2.90 | |  |  |
| clinical tumor size ≥3cm | 0.808 | | 0.84 | | 0.20 | | 3.55 | |  |  |
| muscularis propria  present | 0.984 | | 0.00 | | 0.00 | | - | |  |  |
| **Area under the curve** | | | | | | | | | | |
|  | | area | | std-error | | asymptotic significance | | asymptotic 95% confidence interval | | |
|  |  |  |  |  |  |  |  | lower | | upper |
| ALLICA observer1 (mm) | | .532 | | .088 | | .734 | | .360 | | .704 |
| ALLICA observer2 (mm) | | .510 | | .092 | | .915 | | .329 | | .691 |

Supplementary Table 3: Area under the curve for ROC analysis of ALLICA measurements of both observers for T2 progression


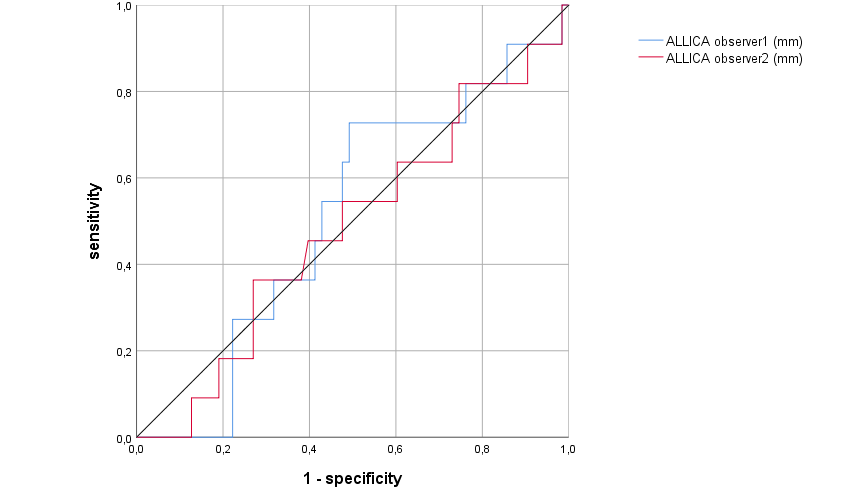


Supplementary figure 1: ROC curve of ALLICA measurement of both observers for T2 progression

| **coordinate table** | | | |
| --- | --- | --- | --- |
|  | threshold^a^ | sensitivity | 1 - specificity |
| observer 1 [mm] | -,8380 | 1,000 | 1,000 |
|  | ,2110 | 1,000 | ,985 |
|  | ,2693 | 1,000 | ,971 |
|  | ,2873 | ,909 | ,971 |
|  | ,2981 | ,909 | ,956 |
|  | ,3151 | ,909 | ,941 |
|  | ,3435 | ,909 | ,926 |
|  | ,3773 | ,909 | ,912 |
|  | ,4073 | ,909 | ,897 |
|  | ,4385 | ,909 | ,882 |
|  | ,4870 | ,909 | ,868 |
|  | ,6115 | ,909 | ,853 |
|  | ,7145 | ,818 | ,853 |
|  | ,7850 | ,818 | ,838 |
|  | ,9265 | ,818 | ,824 |
|  | 1,0280 | ,818 | ,809 |
|  | 1,1710 | ,818 | ,794 |
|  | 1,3650 | ,818 | ,779 |
|  | 1,4850 | ,818 | ,765 |
|  | 1,6475 | ,818 | ,750 |
|  | 1,8030 | ,818 | ,735 |
|  | 1,8795 | ,727 | ,735 |
|  | 1,9860 | ,727 | ,721 |
|  | 2,1115 | ,727 | ,706 |
|  | 2,2700 | ,727 | ,691 |
|  | 2,4030 | ,727 | ,676 |
|  | 2,4930 | ,727 | ,662 |
|  | 2,5800 | ,727 | ,647 |
|  | 2,6425 | ,727 | ,632 |
|  | 2,8350 | ,727 | ,618 |
|  | 3,0720 | ,727 | ,603 |
|  | 3,2225 | ,727 | ,588 |
|  | 3,3580 | ,727 | ,574 |
|  | 3,7220 | ,727 | ,559 |
|  | 4,0915 | ,727 | ,544 |
|  | 4,1795 | ,727 | ,529 |
|  | 4,2390 | ,727 | ,515 |
|  | 4,4290 | ,727 | ,500 |
|  | 4,7013 | ,727 | ,485 |
|  | 4,9463 | ,727 | ,471 |
|  | 5,1695 | ,727 | ,456 |
|  | 5,3232 | ,636 | ,456 |
|  | 5,4723 | ,636 | ,441 |
|  | 5,9182 | ,545 | ,441 |
|  | 6,2805 | ,545 | ,426 |
|  | 6,3897 | ,545 | ,412 |
|  | 6,5698 | ,545 | ,397 |
|  | 6,8370 | ,455 | ,397 |
|  | 7,5650 | ,455 | ,382 |
|  | 8,6760 | ,364 | ,382 |
|  | 9,3360 | ,364 | ,368 |
|  | 9,6780 | ,364 | ,353 |
|  | 10,2445 | ,364 | ,338 |
|  | 10,7300 | ,364 | ,324 |
|  | 11,5550 | ,364 | ,309 |
|  | 13,4040 | ,364 | ,294 |
|  | 16,1205 | ,273 | ,294 |
|  | 17,7720 | ,273 | ,279 |
|  | 18,1345 | ,273 | ,265 |
|  | 18,4210 | ,273 | ,250 |
|  | 18,5360 | ,273 | ,235 |
|  | 18,7800 | ,273 | ,221 |
|  | 19,2604 | ,273 | ,206 |
|  | 19,7394 | ,182 | ,206 |
|  | 19,9524 | ,091 | ,206 |
|  | 20,0254 | ,000 | ,206 |
|  | 20,0870 | ,000 | ,191 |
|  | 20,2110 | ,000 | ,176 |
|  | 20,8971 | ,000 | ,162 |
|  | 22,9011 | ,000 | ,147 |
|  | 26,8345 | ,000 | ,132 |
|  | 29,6730 | ,000 | ,118 |
|  | 32,2885 | ,000 | ,103 |
|  | 35,0020 | ,000 | ,088 |
|  | 37,2259 | ,000 | ,074 |
|  | 40,0359 | ,000 | ,059 |
|  | 41,8630 | ,000 | ,044 |
|  | 44,2110 | ,000 | ,029 |
|  | 63,2437 | ,000 | ,015 |
|  | 81,7464 | ,000 | ,000 |
| observer 2 [mm] | -,9500 | 1,000 | 1,000 |
|  | ,1230 | 1,000 | ,985 |
|  | ,2330 | ,909 | ,985 |
|  | ,3115 | ,909 | ,971 |
|  | ,3565 | ,909 | ,956 |
|  | ,3700 | ,909 | ,941 |
|  | ,4100 | ,909 | ,926 |
|  | ,4500 | ,909 | ,897 |
|  | ,4900 | ,909 | ,882 |
|  | ,5350 | ,818 | ,882 |
|  | ,5850 | ,818 | ,868 |
|  | ,6650 | ,818 | ,853 |
|  | ,7450 | ,818 | ,838 |
|  | ,8450 | ,818 | ,824 |
|  | ,9950 | ,818 | ,809 |
|  | 1,2000 | ,818 | ,794 |
|  | 1,3700 | ,818 | ,779 |
|  | 1,4500 | ,818 | ,765 |
|  | 1,7100 | ,818 | ,750 |
|  | 1,9650 | ,818 | ,735 |
|  | 2,0650 | ,818 | ,721 |
|  | 2,2450 | ,818 | ,706 |
|  | 2,3750 | ,818 | ,691 |
|  | 2,4150 | ,727 | ,691 |
|  | 2,4700 | ,727 | ,676 |
|  | 2,6450 | ,636 | ,676 |
|  | 2,8900 | ,636 | ,662 |
|  | 3,1750 | ,636 | ,647 |
|  | 3,4300 | ,636 | ,632 |
|  | 3,5700 | ,636 | ,618 |
|  | 3,6350 | ,636 | ,603 |
|  | 3,8000 | ,636 | ,588 |
|  | 4,0150 | ,636 | ,574 |
|  | 4,1400 | ,636 | ,559 |
|  | 4,3050 | ,545 | ,559 |
|  | 4,7650 | ,545 | ,544 |
|  | 5,2850 | ,545 | ,529 |
|  | 5,7050 | ,545 | ,515 |
|  | 6,0700 | ,545 | ,500 |
|  | 6,5950 | ,545 | ,485 |
|  | 7,1000 | ,545 | ,471 |
|  | 7,2950 | ,545 | ,456 |
|  | 7,4300 | ,545 | ,441 |
|  | 7,6250 | ,455 | ,441 |
|  | 7,8600 | ,455 | ,426 |
|  | 8,2850 | ,455 | ,412 |
|  | 8,7200 | ,455 | ,397 |
|  | 9,4450 | ,455 | ,382 |
|  | 10,1500 | ,455 | ,368 |
|  | 10,4450 | ,364 | ,353 |
|  | 11,1750 | ,364 | ,338 |
|  | 11,9800 | ,364 | ,324 |
|  | 12,6100 | ,364 | ,309 |
|  | 12,9550 | ,364 | ,294 |
|  | 13,0200 | ,364 | ,279 |
|  | 13,3500 | ,364 | ,265 |
|  | 14,5300 | ,364 | ,250 |
|  | 15,7200 | ,273 | ,250 |
|  | 16,6650 | ,182 | ,250 |
|  | 17,5700 | ,182 | ,235 |
|  | 18,1450 | ,182 | ,221 |
|  | 19,5400 | ,182 | ,206 |
|  | 20,9350 | ,182 | ,191 |
|  | 21,7700 | ,182 | ,176 |
|  | 24,7650 | ,091 | ,176 |
|  | 28,1200 | ,091 | ,162 |
|  | 29,4200 | ,091 | ,147 |
|  | 31,1150 | ,091 | ,132 |
|  | 32,4500 | ,091 | ,118 |
|  | 33,0950 | ,000 | ,118 |
|  | 34,7400 | ,000 | ,103 |
|  | 36,4800 | ,000 | ,088 |
|  | 43,8750 | ,000 | ,074 |
|  | 53,3000 | ,000 | ,059 |
|  | 58,8900 | ,000 | ,044 |
|  | 80,8200 | ,000 | ,029 |
|  | 139,3300 | ,000 | ,015 |
|  | 179,7800 | ,000 | ,000 |
| a. The smallest threshold value is the smallest observed test value minus 1, and the largest threshold value is the largest observed test value plus 1. All other threshold values are the averages of two consecutive, ordered observed test values. | | | |

*Supplementary Table 4: coordinate table for AUC analysis of observer 1 and observer 2 for T2 progression*


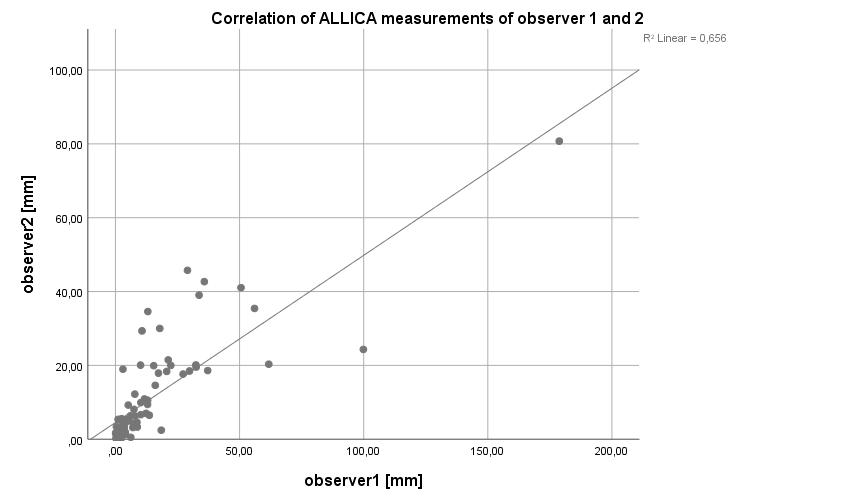


Supplementary figure 2 Correlation analysis of ALLICA measurements of observer 1 and 2
